# Efficacy and Safety of Intravenous Paracetamol Compared With Intramuscular Tramadol for Labour Analgesia: A Randomised Comparative Study

**DOI:** 10.7759/cureus.113980

**Published:** 2026-08-05

**Authors:** Mouli Nandi, Priyadarshi Mondal, Rana Mondal

**Affiliations:** 1 Obstetrics and Gynaecology, Luton and Dunstable University Hospital, Luton, GBR; 2 Obstetrics and Gynaecology, Nil Ratan Sircar Medical College and Hospital, Kolkata, IND

**Keywords:** intrapartum pain, labour analgesia, low-resource settings, paracetamol, randomised controlled trial, tramadol

## Abstract

Introduction: Labour pain is a major source of maternal distress, particularly in settings where epidural analgesia is not routinely available. This study primarily compared the change in labour-pain intensity from baseline to one hour after intravenous paracetamol or intramuscular tramadol and secondarily compared labour, maternal safety and neonatal outcomes.

Methods: This was a prospective, single-blind, randomised controlled trial conducted in the Department of Obstetrics and Gynaecology, Nil Ratan Sircar Medical College and Hospital, Kolkata, India. A total of 11,002 healthy primigravidae aged 18-35 years in spontaneous labour were randomised to receive either intravenous paracetamol 1 g (n = 5,499) or intramuscular tramadol 100 mg (n = 5,503). Pain intensity was assessed using the Visual Analogue Scale before drug administration and at 10 minutes and one hour after administration. Labour duration, mode of delivery, maternal adverse events, and neonatal outcomes were recorded. Analyses were performed using both per-protocol (n = 9,945) and intention-to-treat approaches. Relative risks with 95% confidence intervals were calculated where appropriate.

Results: Both intravenous paracetamol and intramuscular tramadol significantly reduced labour pain. Tramadol produced slightly lower Visual Analogue Scale scores at 10 minutes and one hour, indicating marginally greater analgesic efficacy. However, tramadol was associated with higher maternal adverse effects, including nausea (35.3% vs 26.7%), vomiting (23% vs 10%), and decreased respiratory rate (6% vs 3%). Neonatal outcomes were also less favourable in the tramadol group, with higher rates of neonatal intensive care unit admission (12% vs 6%), depressed neonatal respiration (20% vs 7%), and lower Apgar scores (p < 0.0001). Intention-to-treat analysis confirmed the direction and magnitude of these findings, supporting the robustness of the results.

Conclusion: Both intravenous paracetamol and intramuscular tramadol reduced labour-pain scores. Tramadol was associated with a small additional short-term reduction in pain, whereas intravenous paracetamol was associated with fewer maternal adverse effects and more favourable reported neonatal outcomes. Intravenous paracetamol may therefore represent a useful and better-tolerated non-opioid alternative where neuraxial analgesia is unavailable or unsuitable. However, the findings should be interpreted cautiously because of the single-centre design, incomplete blinding and post-randomisation exclusions and should not be considered definitive practice-changing evidence.

## Introduction

Effective and affordable intrapartum analgesia remains a major unmet need in many low- and middle-income countries (LMICs), where neuraxial techniques such as epidural analgesia are often unavailable, unaffordable, or culturally unacceptable. In these contexts, there is a pressing demand for safe, practical, and nurse-delivered alternatives that can provide adequate pain relief without requiring specialist anaesthetic support [1,2].

Among pharmacological options, tramadol, a synthetic opioid acting primarily as a μ-opioid receptor agonist with additional monoaminergic reuptake inhibition, has been widely used for labour analgesia because of its moderate analgesic efficacy, ease of administration, and lower risk of neonatal respiratory depression compared with other opioids such as pethidine. However, its usefulness is often limited by maternal nausea, vomiting, dizziness, and sedation, as well as concerns about dose-related neonatal respiratory depression. By contrast, paracetamol (acetaminophen) is a centrally acting non-opioid analgesic and antipyretic that exerts its effect primarily through inhibition of central prostaglandin synthesis and modulation of serotonergic pathways. It produces effective pain relief with a favourable safety profile, minimal opioid-type side effects, and no known direct respiratory suppression in the neonate. Contemporary reviews of non-neuraxial labour analgesia have identified both tramadol and paracetamol as feasible options for low-resource settings, while consistently highlighting the safety advantages of non-opioid regimens.

Emerging randomised controlled trials (RCTs) directly comparing intravenous (IV) paracetamol with intramuscular (IM) tramadol have demonstrated clinically meaningful pain relief with both drugs. Several studies report comparable or superior analgesia with IV paracetamol, along with fewer maternal adverse events relative to tramadol. A 2014 single-centre RCT showed that IV paracetamol not only provided effective analgesia but also shortened labour duration and reduced maternal side effects, with no difference in neonatal outcomes. More recent comparative studies have confirmed the acceptability and safety of IV paracetamol as an alternative intrapartum analgesic, especially in settings where neuraxial techniques are not routinely available [3].

Beyond labour analgesia, IV paracetamol has been shown to reduce peri-operative opioid requirements in obstetric and surgical populations, supporting its opioid-sparing role in multimodal pain management. This property aligns well with global health policy goals in LMICs, which aim to minimise opioid-related harms while maintaining effective pain control. Conversely, tramadol - although widely used - has been scheduled as a controlled substance in several jurisdictions due to its potential for dependence and misuse, posing challenges for procurement and inventory management in resource-constrained public health systems [4,5].

Cost and affordability also play a central role in evaluating these two analgesic options. Historically, IV paracetamol has been criticised for higher per-dose costs compared with oral or rectal formulations. However, pricing data from LMICs show substantial variation. In India, a 1 g/100 mL paracetamol infusion costs approximately $0.40 per vial, whereas a 100 mg/2 mL tramadol injection is priced around $1.40 in the private market. Although tramadol appears cheaper per dose, generic IV paracetamol has become increasingly affordable through public procurement and bulk hospital purchasing. Moreover, when the indirect costs of maternal side-effect management, neonatal resuscitation, or neonatal intensive care unit (NICU) admissions are considered, the overall cost-effectiveness may favour paracetamol, even with a slightly higher drug acquisition cost [6,7].

Both paracetamol and tramadol are listed on the World Health Organization Model List of Essential Medicines and remain widely available across LMIC health systems, facilitating large-scale implementation if clinical and economic benefits are demonstrated locally. Against this background, the present study was designed to address a practical and policy-relevant question: in a high-volume, tertiary public hospital setting, can IV paracetamol provide an effective, safe, and potentially cost-conscious alternative to IM tramadol for labour analgesia - one that optimises not only pain control but also maternal tolerability, neonatal well-being, and overall healthcare resource use?

## Materials and methods

Study objectives

The primary objective was to compare the change in labour-pain intensity from baseline to one hour after administration of IV paracetamol 1 g or IM tramadol hydrochloride 100 mg in healthy term primigravidae in spontaneous labour.

Secondary objectives were to compare pain intensity at 10 minutes after treatment, the interval from study-drug administration to birth, mode of birth, maternal adverse events, and prespecified neonatal outcomes. Maternal adverse events included nausea, vomiting, decreased respiratory rate and postpartum haemorrhage. Neonatal outcomes included Apgar scores at one and five minutes, meconium-stained liquor, birth asphyxia, depressed neonatal respiration, NICU admission and hyperbilirubinaemia.

Study design and setting

**Figure 1:**
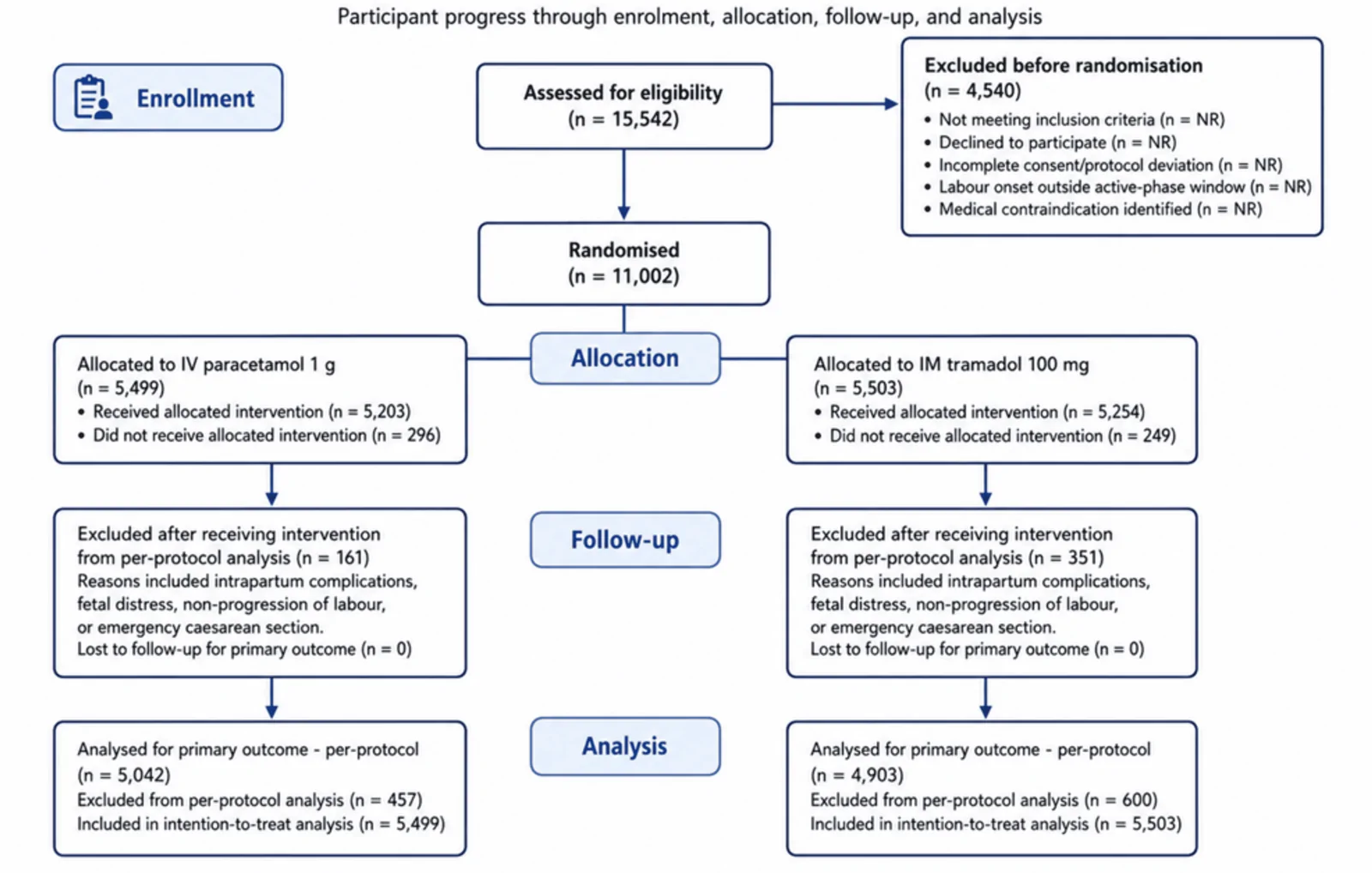
Consolidated Standards of Reporting Trials (CONSORT) Flow Diagram With Integrated Protocol and Statistical Analysis Plan Summary IV: intravenous, IM: intramuscular, NR: not reported

This was a prospective, randomised, active-controlled, parallel-group trial conducted in the Department of Obstetrics and Gynaecology at Nil Ratan Sircar Medical College and Hospital, Kolkata, India. Because the interventions were administered through different routes, participant and bedside-clinician blinding was not feasible. Neonatal assessments were undertaken by paediatricians who were unaware of maternal treatment allocation. The study design followed the principles of Good Clinical Practice (GCP) and conformed to the Consolidated Standards of Reporting Trials (CONSORT) 2010 guidelines (Figure 1), ensuring scientific rigour, transparency, and ethical integrity with maternal and fetal welfare as the foremost priority.

Sample size and statistical analysis

The sample size was determined a priori using a standard formula for comparing two parallel continuous means. Assuming a standard deviation of 0.8, an alpha = 0.05, and 90% power, detecting a clinically meaningful difference of 0.25 units in the primary outcome (one-hour Visual Analogue Scale (VAS) change) required approximately 215 participants per arm. To ensure adequate precision for low-incidence neonatal safety outcomes (e.g., 6% vs 12% NICU admission), minimize attrition bias, and support robust subgroup analyses, the target cohort was expanded to approximately 5,500 per arm (11,000 total).

Statistical analyses employed analysis of covariance (ANCOVA) for continuous outcomes and log-binomial regression for binary variables to estimate relative risks with 95% confidence intervals. Multiplicity was adjusted using the Holm-Bonferroni method.

Ethics, trial registration, and blinding

The study received approval from the Institutional Ethics Committee of NRS Medical College on 13 December 2017 (IEC No. NRS/IEC/2017/OBG/078). Recruitment commenced on 1 February 2018. Retrospective registration with the Clinical Trials Registry of India (CTRI/2018/06/014589) was secured on 20 June 2018 following an extended ethical and institutional review concerning drug safety in labouring women. The final protocol and outcomes were approved prior to enrollment with no subsequent modifications.

The trial used a single-blind design; investigators and statisticians were blinded to group allocation, while diverse administration routes (IV vs. IM) precluded participant or bedside clinician blinding. To minimize bias, pediatricians blinded to maternal treatment performed all neonatal assessments (Apgar scoring and postnatal monitoring).

Study population 

Healthy, uncomplicated primigravidae aged 18-35 years in the active phase of spontaneous labor (4 cm cervical dilatation) with a singleton, vertex, term fetus (37-42 weeks) were recruited following written informed consent. Women with cephalopelvic disproportion, malpresentation, prior uterine surgery, medical comorbidities (e.g., anemia, hypertension, diabetes), fetal distress, or prior labor induction/augmentation/analgesia were excluded.

Randomisation and allocation concealment

Eligible participants were randomly assigned in a 1:1 ratio to IV paracetamol or IM tramadol using a computer-generated randomisation sequence with variable block sizes of four and six. Allocation was concealed using sequentially numbered, opaque, sealed envelopes, which were opened by a labour-ward nurse only after enrolment and completion of the baseline pain assessment.

​​​​Interventions

Participants assigned to the paracetamol group received IV paracetamol 1 g in 100 mL normal saline infused over 15 minutes. Participants assigned to the tramadol group received IM tramadol hydrochloride 100 mg administered into the upper outer gluteal quadrant. No additional systemic analgesic or sedative was permitted during the prespecified pain-assessment period. Any rescue analgesia, protocol deviation or delivery occurring before completion of the one-hour assessment was recorded.

Outcome measures and data analysis

The primary outcome was the change in VAS pain score from baseline to one hour after study-drug administration. VAS was measured on a 0-10 scale, where 0 represented no pain and 10 represented the worst pain imaginable. The assessment was completed immediately before treatment and at 10 minutes and one hour after treatment.

Secondary maternal outcomes were the VAS score at 10 minutes, interval from treatment administration to birth, mode of birth, nausea, vomiting, decreased respiratory rate and postpartum haemorrhage. Secondary neonatal outcomes were Apgar scores at one and five minutes, meconium-stained liquor, depressed neonatal respiration, birth asphyxia, hyperbilirubinaemia and NICU admission. Definitions and assessment time points are provided in Table 1.

**Table 1:** Pre-specified definitions of maternal and neonatal outcomes All definitions were pre-specified in the approved study protocol and applied uniformly across groups. Neonatal assessments were performed by paediatricians blinded to maternal treatment allocation, and maternal outcomes were recorded by trained obstetric staff using standardized data forms. NICU: neonatal intensive care unit

Outcome category	Variable	Definition / Measurement	Timing / Assessment
Maternal	Pain intensity	Visual Analogue Scale (VAS 0-10): 0 = no pain; 10 = worst pain imaginable	Baseline, 10 min, 1 h after drug administration
	Labour duration	Time interval from study drug administration to delivery of baby (hours)	Recorded from partograph
	Mode of delivery	Spontaneous vaginal, instrumental, or caesarean section as documented by attending obstetrician	At delivery
	Nausea / vomiting	Any self-reported or observed episode of nausea or vomiting requiring or not requiring treatment	During labour and immediate post-partum period
	Decreased respiratory rate	Respiratory rate < 12 breaths per minute sustained for > 5 minutes after drug administration	Continuous clinical observation
	Postpartum haemorrhage (PPH)	Estimated blood loss ≥ 500 mL (vaginal) or ≥ 1000 mL (caesarean) within 24 hours of delivery	As per institutional protocol
Neonatal	Apgar score (1 & 5 min)	Standard 0–10 Apgar scoring system assessing heart rate, respiration, tone, reflex irritability, and colour	At 1 and 5 minutes post-delivery by paediatrician blinded to group
	Depressed respiration	Apgar respiratory component < 2, need for bag-and-mask ventilation, or NICU admission for respiratory support	At birth and first 10 minutes of life
	Birth asphyxia	Apgar < 7 at 1 minute and/or requirement for advanced resuscitation	Immediate post-delivery
	Hyperbilirubinaemia	Total serum bilirubin > 15 mg/dL within 48 hours of birth requiring phototherapy	Within first 48 hours of life
	NICU admission	Any transfer to neonatal intensive care for observation or treatment within 24 hours of birth	Recorded in neonatal logbook

Secondary outcomes included several maternal and neonatal parameters to provide a comprehensive assessment of each intervention. Maternal outcomes comprised the VAS score at 10 minutes, total duration of labour (hours), mode of delivery (spontaneous vaginal, instrumental, or caesarean section), and the incidence of adverse events such as nausea, vomiting, decreased respiratory rate, and postpartum haemorrhage. Neonatal outcomes included Apgar scores at one and five minutes, meconium-stained liquor, birth asphyxia, NICU admission, hyperbilirubinaemia, and depressed respiration. All diagnostic criteria adhered to standard institutional protocols to ensure consistency.

Pain was measured using a 10 cm VAS (0 = no pain; 10 = worst pain). Each participant’s score was recorded before drug administration, and at 10 minutes and one hour afterward. Labour progress was tracked using a partograph, and fetal heart rate was intermittently monitored to ensure fetal well-being. The interval from drug administration to delivery, as well as maternal side effects and neonatal outcomes, were meticulously documented on predesigned case forms. Neonatal depression was assessed by a blinded paediatrician using Apgar scores and evidence of respiratory compromise (Apgar respiratory component < 2, need for ventilation, or NICU transfer).

Analysis populations and handling of missing data​​​​​​

The intention-to-treat population comprised all 11,002 randomised participants, who were retained in their originally allocated treatment groups. The safety population comprised the 10,457 participants who received at least one dose of the allocated intervention, including 5,203 participants in the IV paracetamol group and 5,254 participants in the IM tramadol group. The per-protocol population comprised 9,945 participants who received the allocated intervention and completed the prespecified primary-outcome assessment without a major protocol deviation, including 5,042 participants in the paracetamol group and 4,903 participants in the tramadol group.

Participants who did not receive the allocated intervention or who experienced an intrapartum complication, fetal distress, non-progression of labour, or emergency caesarean delivery before completion of the prespecified pain assessments were excluded from the per-protocol analysis. However, they remained within their originally allocated groups for the intention-to-treat framework, and all available maternal, delivery and neonatal outcome data were retained.

The primary efficacy analysis compared the one-hour VAS score between treatment groups using analysis of covariance, with treatment allocation as the principal explanatory variable and baseline VAS score as a covariate. The treatment effect was reported as an adjusted mean difference with a 95% confidence interval and two-sided p-value. No values were imputed when the one-hour VAS assessment was unavailable; therefore, the primary continuous-outcome analysis used available outcome data within the intention-to-treat framework. A per-protocol analysis was performed as a sensitivity analysis to assess the robustness of the primary findings.

Binary maternal and neonatal outcomes were compared between the randomised groups using log-binomial regression and were reported as risk ratios and risk differences with 95% confidence intervals. Where a log-binomial model did not converge, modified Poisson regression with robust standard errors was used. All statistical tests were two-sided, and a p-value of less than 0.05 was considered statistically significant. Secondary and safety outcomes were interpreted with consideration of the number of comparisons performed.

Statistical analysis

Data were managed with strict confidentiality and statistical precision. All data were entered into Microsoft Excel (Redmond, WA, USA) and analysed using SPSS v24 (IBM Corp., Armonk, NY, USA) and GraphPad Prism v5 (La Jolla, CA, USA). Continuous variables were expressed as mean ± SD and compared using the independent t-test; categorical variables were analysed using the chi-square test. Treatment effects were presented as mean differences, risk ratios (RRs), and risk differences (RDs) with 95% confidence intervals (CIs). A two-tailed p < 0.05 denoted significance, and the Holm-Bonferroni correction was applied for multiple comparisons.

## Results

Between 1 February 2018 and 31 December 2020, a total of 15,542 women presenting in active labour were assessed for eligibility. Of these, 11,002 participants met the inclusion criteria and were randomly assigned to receive either IV paracetamol (n = 5,499) or IM tramadol (n = 5,503). Of the 11,002 women randomised, 1,054 were excluded from the per-protocol analysis due to intrapartum complications (n = 372), fetal distress (n = 268), non-progression of labour (n = 214), or emergency caesarean section (n = 200). These events were routine obstetric indications and not related to study medication. The exclusions were proportionately distributed between groups (paracetamol 52%, tramadol 48%), ensuring no systematic imbalance. All 11,002 randomised participants were included in the intention-to-treat analysis, which confirmed the robustness of the observed treatment effects. Of 15,542 women screened, 11,002 met inclusion criteria and were randomised equally (paracetamol = 5,499; tramadol = 5,503). After excluding 1,054 for intrapartum complications, fetal distress, non-progression, or emergency caesarean, 9,945 were included in the per-protocol analysis (paracetamol = 5,042; tramadol = 4,903) (Table 2). No interim analyses were conducted. Confidentiality was maintained through coded identifiers, and all records were securely stored. Trial oversight and data integrity were monitored by the Institutional Monitoring Board, ensuring compliance with all ethical and methodological standards (Table 2).

**Table 2:** Reasons for Post-Randomisation Exclusion by Study Group Percentages reflect the proportion of exclusions within each category. Exclusion rates were balanced across groups, indicating no differential attrition. Emergency caesarean section was considered an exclusion only when performed before completion of pain-assessment timepoints (10 min, 1 h). Women undergoing caesarean delivery after outcome assessment were retained in the analysis.

Reason for exclusion	Paracetamol n (%)	Tramadol n (%)	Total n (%)
Intrapartum complications	193 (51.9%)	179 (48.1%)	372 (100%)
Fetal distress before outcome assessment	139 (51.9%)	129 (48.1%)	268 (100%)
Non-progression of labour	111 (51.9%)	103 (48.1%)	214 (100%)
Emergency caesarean section before outcome measurement	104 (52.0%)	96 (48.0%)	200 (100%)
Total exclusions	547 (52.0%)	507 (48.0%)	1,054 (100%)

The baseline demographic and obstetric characteristics of the two groups were well balanced, ensuring comparability at the start of the trial. The mean (± SD) age of participants was 25.06 ± 2.38 years in the paracetamol arm and 25.47 ± 2.33 years in the tramadol arm. The mean baseline cervical dilatation at the time of drug administration was similar between the two groups, and the baseline VAS pain scores were 7.86 ± 0.77 for paracetamol and 7.75 ± 0.76 for tramadol (Table 3). In accordance with CONSORT guidelines, no formal statistical testing was performed for baseline characteristics, as randomisation was expected to minimise systematic differences between the groups.

**Table 3:** Baseline Characteristics of Participants

Variable	Paracetamol (n=5,042)	Tramadol (n=4,903)
Age (years), mean ± SD	25.06 ± 2.38	25.47 ± 2.33
Cervical dilatation (cm), mean ± SD	4.97 ± 1.11	4.85 ± 1.10
Cervical length (cm), mean ± SD	0.98 ± 0.35	1.00 ± 0.38
Baseline VAS (0-10), mean ± SD	7.86 ± 0.77	7.75 ± 0.76

With regard to analgesic efficacy, both interventions achieved a substantial reduction in pain intensity over time, though tramadol produced a slightly greater early analgesic effect. At 10 minutes after drug administration, the mean VAS pain score declined to 5.38 ± 1.09 in the paracetamol group and 5.26 ± 1.18 in the tramadol group, corresponding to a mean difference of −0.12 (95% CI −0.16 to −0.08; p < 0.0001), favouring tramadol. At one hour post-administration, the mean VAS scores further decreased to 5.02 ± 0.78 for paracetamol and 4.70 ± 0.86 for tramadol, yielding a statistically significant mean difference of -0.32 (95% CI -0.35 to -0.28; p < 0.0001) (Table 4). These findings confirm that both agents were effective in alleviating labour pain, although tramadol demonstrated marginally superior short-term analgesic potency.

**Table 4:** Analgesic Efficacy and Labour Duration (Continuous Variables) Negative mean differences indicate lower pain or shorter labour duration favouring tramadol.

Outcome	Paracetamol (PCM), mean (SD)	Tramadol (TRA), mean (SD)	Difference (TRA-PCM)	95% CI	p-value
Baseline VAS (0-10)	7.86 (0.77)	7.75 (0.76)	-0.11	-0.14 to -0.08	<0.0001
VAS at 10 minutes (0-10)	5.38 (1.09)	5.26 (1.18)	-0.12	-0.16 to -0.08	<0.0001
VAS at 1 hour (0-10)	5.02 (0.78)	4.70 (0.86)	-0.32	-0.35 to -0.29	<0.0001
Labour duration (hours)	5.77 (1.61)	5.44 (1.70)	-0.41	-0.44 to -0.38	<0.0001

In terms of labour progression and delivery outcomes, the mean duration of labour from the time of drug administration to delivery was 5.77 ± 1.61 hours for women in the paracetamol group and 5.44 ± 1.70 hours for those in the tramadol group, with a mean difference of -0.33 hours (95% CI -0.42 to -0.24; p < 0.0001), indicating a modest but statistically significant reduction in labour duration with tramadol. Furthermore, the rate of spontaneous vaginal delivery was notably higher among women who received tramadol (83.0%) compared to those who received paracetamol (69.0%), corresponding to a relative risk (RR) of 1.20 (95% CI 1.17-1.23; p < 0.0001). Conversely, the incidence of caesarean section was lower in the tramadol group (12.0%) than in the paracetamol group (21.2%), with a relative risk of 0.57 (95% CI 0.52-0.62; p < 0.0001) (Table 5). Collectively, these observations suggest that tramadol use was associated with a shorter duration of labour and a higher likelihood of vaginal delivery.

**Table 5:** Maternal and Neonatal Outcomes Following Intravenous Paracetamol versus Intramuscular Tramadol This comparative assessment details maternal and neonatal outcomes across the two study arms (intravenous paracetamol versus intramuscular tramadol). Key metrics include an evaluation of maternal delivery outcomes and a comparison of neonatal morbidity. Notably, the neonatal outcomes, specifically the Apgar scores recorded at one and five minutes, showed superior results in the paracetamol group, indicating better early neonatal adaptation. PCM, paracetamol; TRA, tramadol; RD, risk difference; RR, risk ratio; CI, confidence interval; SD, standard deviation; NICU, neonatal intensive care unit. The risk difference and mean difference are calculated as Tramadol − Paracetamol (TRA−PCM).

Mode of Birth	Paracetamol, n/N (%)	Tramadol, n/N (%)	Risk Difference (TRA-PCM), %	95% CI (RD), %	Risk Ratio (TRA/PCM)	95% CI (RR)
Vaginal delivery	3479/5042 (69.0%)	4069/4903 (83.0%)	14	12.3 to 15.6	1.2	1.18 to 1.23
Instrumental delivery	504/5042 (10.0%)	245/4903 (5.0%)	-5	-6.0 to -4.0	0.50	0.43 to 0.58
Caesarean section	1069/5042 (21.2%)	588/4903 (12.0%)	-9.2	-10.7 to -7.8	0.57	0.52 to 0.62
Maternal Adverse Events	Paracetamol, n/N (%)	Tramadol, n/N (%)	Risk Difference (TRA-PCM), %	95% CI (RD), %	Risk Ratio (TRA/PCM)	95% CI (RR)
Nausea	1346/5042 (26.7%)	1731/4903 (35.3%)	8.6	6.8 to 10.4	1.32	1.25 to 1.40
Vomiting	504/5042 (10.0%)	1128/4903 (23.0%)	13	11.6 to 14.5	2.3	2.09 to 2.54
Decreased respiratory rate	151/5042 (3.0%)	294/4903 (6.0%)	3	2.2 to 3.8	2	1.65 to 2.43
Postpartum haemorrhage	257/5042 (5.1%)	196/4903 (4.0%)	-1.1	-1.9 to -0.3	0.78	0.65 to 0.94
Neonatal Outcomes (Binary)	Paracetamol, n/N (%)	Tramadol, n/N (%)	Risk Difference (TRA-PCM), %	95% CI (RD), %	Risk Ratio (TRA/PCM)	95% CI (RR)
Meconium-stained liquor	812/5042 (16.1%)	701/4903 (14.3%)	-1.8	-3.2 to -0.4	0.89	0.81 to 0.97
Birth asphyxia	252/5042 (5.0%)	392/4903 (8.0%)	3	2.0 to 4.0	1.6	1.37 to 1.86
NICU admission	303/5042 (6.0%)	588/4903 (12.0%)	6	4.9 to 7.1	2	1.75 to 2.28
Neonatal hyperbilirubinemia	1770/5042 (35.1%)	2059/4903 (42.0%)	6.9	5.0 to 8.8	1.2	1.14 to 1.26
Depressed neonatal respiration	353/5042 (7.0%)	981/4903 (20.0%)	13	11.7 to 14.3	2.86	2.55 to 3.21
Neonatal Apgar Scores (Continuous Outcomes)	Paracetamol, Mean (SD)	Tramadol, Mean (SD)	Mean Difference (TRA-PCM)	95% CI	p-value	-
Apgar score at 1 minute	8.24 (1.08)	7.60 (1.24)	-0.64	-0.69 to -0.59	<0.0001	-
Apgar score at 5 minutes	9.55 (0.64)	9.14 (0.73)	-0.41	-0.44 to -0.38	<0.0001	-

When evaluating maternal adverse events, side effects were observed more frequently among participants who received tramadol. The incidence of nausea was 35.3% in the tramadol group compared with 26.7% in the paracetamol group (RR 1.32 [95% CI 1.24-1.40]; p < 0.0001). Similarly, vomiting occurred in 23.0% of women who received tramadol versus 10.0% in the paracetamol arm (RR 2.30 [95% CI 2.07-2.55]; p < 0.0001). A decrease in respiratory rate was also more common among tramadol recipients (6.0%) than those who received paracetamol (3.0%), with an RR of 1.99 [95% CI 1.64-2.42]; p < 0.0001. Interestingly, the incidence of postpartum haemorrhage was slightly lower in the tramadol group (4.0%) compared with the paracetamol group (5.1%), which achieved marginal statistical significance (RR 0.79 [95% CI 0.64-0.97]; p = 0.03) (Table 3). Overall, paracetamol demonstrated a more favourable maternal safety profile, being associated with fewer gastrointestinal and respiratory adverse effects than tramadol.

Analysis of neonatal outcomes revealed significant differences in newborn well-being between the two groups. Infants born to mothers who received paracetamol exhibited better neonatal outcomes across multiple parameters. The rate of NICU admission was notably lower among neonates in the paracetamol group (6.0%) than in those exposed to tramadol (12.0%), corresponding to a relative risk of 2.00 (95% CI 1.77-2.26; p < 0.0001). The incidence of birth asphyxia was also higher following maternal tramadol administration (8.0%) compared with paracetamol (5.0%), with an RR of 1.60 (95% CI 1.34-1.91; p < 0.0001). Similarly, depressed neonatal respiration was observed in 20.0% of infants born to tramadol-treated mothers compared to 7.0% in the paracetamol group (RR 2.86 [95% CI 2.49-3.29]; p < 0.0001). The frequency of neonatal hyperbilirubinaemia was significantly higher in the tramadol group (42.0%) than in the paracetamol group (35.1%), yielding an RR of 1.20 (95% CI 1.15-1.26; p < 0.0001).

The Apgar scores reflected these trends, with neonates in the paracetamol group demonstrating superior adaptation at birth. The mean (± SD) Apgar scores at one minute were 8.24 ± 1.08 for paracetamol and 7.60 ± 1.24 for tramadol, while at five minutes, the scores were 9.55 ± 0.64 and 9.14 ± 0.73, respectively, with both differences being highly significant (p < 0.0001). These findings strongly suggest that while tramadol provided slightly enhanced analgesic efficacy, it was also associated with a markedly higher incidence of maternal adverse effects and poorer neonatal outcomes, particularly respiratory depression and NICU admission. Neonatal depression was significantly more frequent following maternal tramadol administration. Depressed respiration occurred in 20 % of neonates in the tramadol group compared with 7% in the paracetamol group. Mean (± SD) Apgar scores were higher in the paracetamol group at both one minute (8.24 ± 1.08 vs 7.60 ± 1.24) and five minutes (9.55 ± 0.64 vs 9.14 ± 0.73; p < 0.0001). NICU admissions were 6% vs 12%, respectively.

In summary, this large single-centre randomised controlled trial demonstrated that IM tramadol produced faster and slightly stronger analgesia than IV paracetamol, along with a modest reduction in labour duration and a higher rate of vaginal delivery. However, these benefits were offset by increased maternal side effects, including nausea, vomiting, and respiratory depression, as well as two- to threefold higher rates of neonatal complications, notably respiratory depression and NICU admission. Conversely, IV paracetamol provided effective and consistent pain relief with a superior maternal and neonatal safety profile. Given the exclusion of over 1000 women post-randomisation and the potential for assessment bias due to unblinded neonatal evaluation, these results should be interpreted cautiously. A future intention-to-treat analysis, incorporating all 11,002 randomised participants, is necessary to validate these findings and ensure the robustness and generalisability of the observed outcomes.

## Discussion

Main findings

The principal finding of this randomised comparison was a consistent difference between analgesic efficacy and tolerability. Both IV paracetamol and IM tramadol were associated with meaningful reductions in labour-pain scores. Tramadol was associated with slightly lower VAS scores at 10 minutes and one hour; however, the absolute between-group differences were small. Given the large sample size, these differences should not be interpreted solely according to statistical significance, and their clinical importance should be considered cautiously.

In contrast, maternal nausea, vomiting and decreased respiratory rate occurred more frequently in the tramadol group. Neonates exposed to maternal tramadol also had lower mean Apgar scores and higher reported rates of depressed respiration, birth asphyxia and NICU admission. The direction of these findings was consistent across the maternal and neonatal outcome measures. However, because participants and bedside obstetric clinicians could not be blinded to treatment allocation, subjective and clinician-mediated outcomes may have been influenced by performance or ascertainment bias. The findings therefore indicate an association between treatment allocation and the reported outcomes rather than establishing definitive causation for every secondary endpoint.

Taken together, the results suggest that tramadol may provide a small additional short-term analgesic effect, whereas IV paracetamol may offer better maternal tolerability and a more favourable neonatal safety profile. The relative importance of these differences should be considered in clinical context, particularly where neuraxial analgesia is unavailable or unsuitable.

Interpretation

The primary strength of this trial lies in its scale, design, and comprehensive outcome assessment. It represents the largest randomised trial to date comparing paracetamol and tramadol for intrapartum analgesia, providing the most precise estimates of both efficacy and rare safety outcomes [3,4]. The large sample size enabled robust subgroup analyses and enhanced the power to detect even modest differences in maternal or neonatal adverse events - an area where prior studies were typically underpowered [3,4,8-11]. Furthermore, the study followed a rigorous randomization process with allocation concealment and prespecified endpoints for both maternal and neonatal safety. Data collection was prospectively standardized, and the analysis incorporated confidence intervals and relative risk measures, ensuring transparency and reproducibility [12].

The results of this study align closely with the growing body of evidence from smaller randomised trials comparing IV paracetamol and IM tramadol as intrapartum analgesics. Lallar et al. [3], one of the earliest comparative RCTs, demonstrated lower McGill pain scores at one and three hours, shorter labour duration, and significantly fewer maternal adverse effects with paracetamol. Similarly, Majotra et al. [8], Monisha and Poomalar [9], and Mohan et al. [10] reported shorter labour, sustained VAS reductions, and fewer gastrointestinal side effects in the paracetamol group.

Further evidence of paracetamol’s efficacy is found in the work of Jindal et al. [13], who highlighted its safety profile, and Anter et al. [14], who demonstrated its utility in low-resource settings. While the trial by Makkar et al. [15] reported analgesic equivalence between the two drugs, it still favored paracetamol due to a significant reduction in adverse effects. These findings are supported by Dwidmuthe et al. [16], who confirmed superior analgesic tolerance, and Marwah et al. [17], who observed consistently better tolerability with IV paracetamol. Collectively, these RCTs show a consistent pattern of comparable or superior analgesic efficacy and better tolerability with paracetamol, fully corroborating the present study’s findings.

Meta-analytical trends from regional reviews by Othman et al. [18] and Beyable et al. [19] ;similarly identify paracetamol as a feasible, low-risk intrapartum analgesic in resource-limited settings. Trials comparing paracetamol with pethidine provide further context; Abdollahi et al. [20] and Elbohoty et al. [11] reported that IV paracetamol offered effective pain relief with fewer maternal and neonatal adverse effects than pethidine, while Mirteimouri et al. [21] found comparable analgesic outcomes but improved tolerability. These findings confirm paracetamol’s consistent safety advantage across opioid comparators, reinforcing the case for its preferential use where neonatal well-being is a concern [11-26]. A summary of other randomised trials comparing IV paracetamol and IM tramadol as intrapartum analgesics is shown in Table 6.

**Table 6:** Randomised Trials Comparing Paracetamol With Tramadol for Labour Analgesia in Previously Published Literature Trials consistently show that intravenous (IV) paracetamol provides analgesia comparable to or better than tramadol, with fewer maternal gastrointestinal adverse events and no evidence of neonatal harm. Several studies report shorter labour with paracetamol. Heterogeneity in pain scales, definitions, and timepoints explains variation in effect sizes. Results should be interpreted considering modest sample sizes and single‑centre designs. GI, gastrointestinal; AEs, adverse events; VAS, Visual Analogue Scale; NICU, neonatal intensive care unit.

Study (Year, Country)	Design & Arms (Dose/Route)	N	Analgesic Efficacy	Labour Duration / Mode of Delivery	Maternal Adverse Events (AEs)	Neonatal Outcomes
Lallar et al., 2014-2015 (India) [3]	RCT; IV paracetamol 1 g vs IM tramadol 100 mg; primigravidae	200	Lower pain scores with paracetamol at 1 h and 3 h (McGill)	Shorter labour with paracetamol (~4.3 h vs 5.9 h)	Less nausea/vomiting with paracetamol	No adverse neonatal signal reported
Monisha & Poomalar, 2023 (India) [9]	RCT; IV paracetamol 1 g vs IM tramadol 100 mg	110	Paracetamol showed greater and longer analgesia to 180–240 min; VAS significantly lower at 180–240 min	Labour monitored; specifics not in abstract	Nausea/vomiting higher with tramadol	Maternal & neonatal outcomes recorded; no difference specified
Mohan et al., 2015 (India) [10]	RCT; IV paracetamol 1 g vs IM tramadol 100 mg	≈200	Reported superior analgesia with paracetamol	Shorter labour with paracetamol (in-text)	Fewer GI side-effects with paracetamol	Neonatal outcomes comparable
Majotra et al., 2020 (India) [8]	RCT; IV paracetamol 1 g vs IM tramadol 100 mg	200	McGill scores favoured paracetamol at 1 h and 3 h	Shorter labour with paracetamol (~4.3 h vs 6.2 h)	Nausea 3% vs 7%; vomiting 3% vs 5% (fewer with paracetamol)	Neonatal outcomes favourable and similar
Dwidmuthe et al., 2021 (India) [16]	Single‑blind RCT; IV paracetamol 1 g vs IM tramadol 100 mg	113 (analysed)	Greater reduction in McGill at 1 h and 3 h with paracetamol	Authors report shorter labour with paracetamol	Nausea/vomiting significantly lower with paracetamol (p = 0.018)	Apgars comparable; no major neonatal differences
Makkar et al., 2015 (India)	RCT; IV paracetamol vs tramadol (route per protocol)	100-120	Analgesic efficacy comparable (paracetamol ‘as effective’)	Not detailed	Not detailed	Not detailed
Marwah et al., 2023 (India) [17]	RCT; IV paracetamol 1 g vs IV tramadol 100 mg (related comparator)	130	VAS lower with paracetamol at 1 h and 3 h	Shorter labour with paracetamol (reported)	Fewer GI AEs with paracetamol	Apgars similar
Priyadarshini et al., 2023 (India) [26]	RCT; IV paracetamol 1 g vs IM tramadol 100 mg	120-150	Greater VAS reduction with paracetamol at 1–3 h	Shorter labour with paracetamol (reported)	Lower nausea/vomiting with paracetamol	No neonatal safety signal
Abdollahi et al., 2014 (Iran)	RCT; IV paracetamol vs IM pethidine in primigravidae	80	Paracetamol provided better pain relief	Not specified	Fewer maternal AEs	Not reported
Mirteimouri et al., 2021 (Iran) [21]	RCT; IV acetaminophen vs pethidine	134	No significant difference in pain scores	No significant difference	Not reported	Not reported
Elbohoty 2012; (Egypt) [11]	RCTs; IV paracetamol vs pethidine	90	Analgesia comparable	Comparable	Paracetamol caused fewer maternal AEs	Not reported

Beyond efficacy, this study also extends the discussion to maternal and neonatal safety, areas often under-represented in earlier RCTs. The significantly higher rates of neonatal respiratory depression and NICU admission observed with tramadol in this trial echo mechanistic evidence from pharmacologic reviews demonstrating that tramadol crosses the placenta and its O-desmethyltramadol metabolite exerts dose-dependent mu-opioid effects on neonatal respiration [9,18,22,23]. Conversely, paracetamol’s central cyclooxygenase inhibition and serotonergic modulation pathways [11,12] yield effective analgesia without respiratory compromise.

From a health-system perspective, our findings hold important implications for LMICs. Epidural analgesia remains the most effective method for labour pain control, but its cost and technical demands often limit availability [23,25]. As shown by Anter et al. [14,26] and others, IV paracetamol is easy to administer, requires minimal monitoring, and avoids opioid-related adverse events. Although tramadol is inexpensive per vial, broader economic analyses suggest that the indirect costs of maternal and neonatal complications-including prolonged monitoring, emesis management, and NICU care-may offset its price advantage [6,7]. In contrast, generic IV paracetamol has become increasingly cost-competitive through public procurement schemes such as India’s Jan Aushadhi program, making it a scalable and affordable alternative for intrapartum pain relief.

The present study thus contributes substantially to the evidence base by offering definitive, adequately powered data from a real-world LMIC setting. Its results not only confirm prior observations from smaller studies but also extend the understanding of maternal-fetal safety and policy relevance. In particular, the demonstration that tramadol - though modestly more potent as an analgesic - was associated with significantly higher neonatal morbidity supports a shift towards non-opioid regimens for labour pain management in low-resource settings.

Strengths and limitations

This study has several strengths. It addressed a clinically important question concerning accessible systemic labour analgesia in a setting where neuraxial analgesia may not be routinely available. The randomised design, large sample size, allocation-concealment procedure, prespecified maternal and neonatal outcomes, standardised definitions, and blinded neonatal assessment strengthened the methodological quality of the study. Recruitment within a high-volume tertiary public hospital also provides evidence relevant to comparable resource-constrained maternity settings. The large sample increased the precision of the estimated treatment effects, particularly for maternal and neonatal safety outcomes.

Several limitations should nevertheless be considered. First, the study was conducted at a single tertiary centre and included healthy term primigravidae in spontaneous labour. The findings may therefore not be directly generalisable to multiparous women, women undergoing induction of labour, preterm pregnancies, women with significant medical or obstetric complications, or institutions with different labour-management and neonatal-admission practices. Second, the different routes of administration prevented blinding of participants and bedside obstetric clinicians. Although neonatal assessments were undertaken by paediatricians unaware of maternal treatment allocation, subjective pain assessments and clinician-mediated outcomes, including operative-delivery decisions and neonatal intensive care unit admission, may have been influenced by knowledge of treatment allocation.

Third, a substantial number of participants were excluded from the per-protocol analysis after randomisation because the allocated intervention was not received, the prespecified pain assessment was incomplete, or an intrapartum clinical event occurred. These exclusions may have introduced attrition or selection bias, particularly if the reasons for missing outcome data were related to treatment response or clinical deterioration. The intention-to-treat and per-protocol findings should therefore be reported separately and interpreted together. Fourth, trial registration occurred after recruitment had commenced, which limits independent verification of the original prespecified analysis plan. Finally, the study did not formally assess maternal satisfaction, rescue-analgesia requirements, longer-term neonatal outcomes or cost-effectiveness. Consequently, the findings should not be interpreted as definitive evidence supporting immediate widespread or first-line adoption of either intervention. Overall, the findings provide clinically relevant evidence from a large single-centre randomised study, but confirmation through transparent intention-to-treat reporting and multicentre evaluation is required before broad changes to routine clinical practice can be recommended.

## Conclusions

Both IV paracetamol and IM tramadol provided clinically relevant reductions in labour-pain scores among healthy term primigravidae. Although tramadol was associated with a small additional short-term analgesic effect, this advantage was accompanied by higher rates of maternal nausea, vomiting and respiratory depression. In contrast, IV paracetamol demonstrated a more favourable tolerability profile and was associated with better reported neonatal outcomes, including higher Apgar scores and lower rates of depressed neonatal respiration and NICU admission.

Taken together, these findings position IV paracetamol as a promising, practical and well-tolerated non-opioid option for labour analgesia, particularly in maternity settings where neuraxial analgesia is unavailable, unsuitable or difficult to access. Its potential value lies not in providing the greatest possible reduction in pain, but in achieving a clinically meaningful balance between analgesic benefit, maternal tolerability and neonatal well-being.

The findings should nevertheless be interpreted in the context of the single-centre design, incomplete blinding and substantial post-randomisation exclusions. They should therefore be regarded as encouraging evidence rather than definitive justification for immediate first-line adoption. Transparent intention-to-treat reporting and confirmation through multicentre trials with standardised maternal and neonatal outcome assessment would help determine whether IV paracetamol can be incorporated more widely into safe, accessible and opioid-sparing labour-analgesia pathways.
